# Influenza surveillance in pigs: balancing act between broad diagnostic coverage and specific virus characterization

**DOI:** 10.1186/s40813-024-00367-9

**Published:** 2024-05-19

**Authors:** Julia Stadler, Sophia Zwickl, Sophie Gumbert, Mathias Ritzmann, Kathrin Lillie-Jaschniski, Timm Harder, Annika Graaf-Rau, Vassilis Skampardonis, Matthias Eddicks

**Affiliations:** 1 Clinic for Swine, Centre for Clinical Veterinary Medicine, Ludwig-Maximilians-Universität München, Oberschleißheim, Germany; 2 CEVA Tiergesundheit, Duesseldorf,, Germany; 3 Institute of Diagnostic Virology, Friedrich-Loeffler-Institut, Greifswald-Insel Riems, Germany; 4 Department of Epidemiology, Biostatistics and Economics of Animal Production, School of Veterinary Medicine, University of Thessaly, 43132 Karditsa, Greece

**Keywords:** Swine influenza a virus, Enzootic infection, Sampling material, Subtyping, Multiplex RT-qPCR, Cross-sectional

## Abstract

**Background:**

Monitoring of infectious diseases on swine farms requires a high diagnostic sensitivity and specificity of the test system. Moreover, particularly in cases of swine influenza A virus (swIAV) it is desirable to include characterization of the virus as precisely as possible. This is indispensable for strategies concerning prophylaxis of swIAV and furthermore, to meet the requirements of a purposeful monitoring of newly emerging swIAV strains in terms of vaccine design and public health. Within the present cross-sectional study, we compared the diagnostic value of group samples (wipes of surfaces with direct contact to mouth/nose, dust wipes, udder skin wipes, oral fluids) to individual samples (nasal swabs, tracheobronchial swabs) for both swIAV identification and characterization. Sampling included different stages of pig production on 25 sow farms with attached nursery considered as enzootically infected with swIAV. Firstly, samples were analyzed for IAV genome and subsequently samples with Ct-values < 32 were subtyped by multiplex RT-qPCR.

**Results:**

Nasal swabs of suckling piglets and nursery pigs resulted in a higher odds to detect swIAV (*p* < 0.001) and to identify swIAV subtypes by RT-qPCR (*p* < 0.05) compared to nasal swabs of sows. In suckling piglets, significant higher rates of swIAV detection could be observed for nasal swabs (*p* = 0.007) and sow udder skin wipes (*p* = 0.036) compared to contact wipes. In the nursery, group sampling specimens were significantly more often swIAV positive compared to individual samples (*p* < 0.01), with exception of the comparison between contact wipes and nasal swabs (*p* = 0.181). However, in general nasal swabs were more likely to have Ct-value < 32 and thus, to be suitable for subtyping by RT-qPCR compared to dust wipes, contact wipes, udder skin wipes and tracheobronchial swabs (*p* < 0.05). Interestingly, different subtypes were found in different age groups as well as in different specimens in the same holding.

**Conclusion:**

Although population-based specimens are highly effective for swIAV monitoring, nasal swabs are still the preferable sampling material for the surveillance of on-farm circulating strains due to significantly higher virus loads. Remarkably, sampling strategies should incorporate suckling piglets and different age groups within the nursery to cover as many as possible of the on-farm circulating strains.

**Supplementary Information:**

The online version contains supplementary material available at 10.1186/s40813-024-00367-9.

## Background

Swine influenza A virus (swIAV) is an important pathogen in swine causing respiratory disease and reproductive disorders [1–3]. Besides its negative impact on animal health, and thus, on the economic benefit of affected swine farms, swIAV is a continuous threat to public health [4, 5]. The capability of swIAVs to overcome the species barrier in combination with genetic shift and drift, due to coinfections with different swIAV subtypes might lead to the emergence of new virus variants with pandemic potential [6–9]. Recent publications indicate an increasing prevalence of swIAV infected swine farms and an extending virus variability in Europe within the last decade [8, 10, 11]. This is particularly caused by the emergence of a new human pandemic H1N1 strain in 2009 and its immediate reverse zoonotic transmission into swine populations worldwide where it reassorted extensively with circulating endemic swIAVs [11–13] with the consequence of needing a more sophisticated diagnosis and strain characterization.

The traditional diagnostic approach to identify swIAV in pig farms includes indirect (ELISA, hemagglutination inhibition, [HI]) or direct (RT-qPCR, virus isolation, next generation sequencing [NGS]) methods [9, 14–19]. Nasal swabs comprise the most common sample material for diagnosis by nucleic acid detection or even isolation, whereas blood samples are suitable for the detection of specific antibodies against swIAV. However, both approaches have their drawbacks. Inherently, serological assays are unsuitable to diagnose acute infections but might be used for retrospective studies. Here they suffer from low specificity as highly varying antigens of distinct lineages lead to cross reactions (false positives) or missing detection (false negatives). Vaccination-induced humoral responses further complicate the interpretation of the test outcome [16, 20–22]. Concerning the detection of acute swIAV infections by RT-qPCR in nasal swabs, the timeframe for the detection is limited to the acute phase which typically lasts between 1 and 7 days after infection [1, 23, 24]. Moreover, virus circulation and patterns of shedding might vary between age or production groups and swIAV strains [23]. Fulminant epidemic outbreaks might require different diagnostic approaches compared to more insidious enzootic scenarios. Thus, an “one size fits all” diagnostic approach is not available to reach the different strata of diagnostic aims in terms of swIAV.

Concerning swIAV diagnosis, different levels of health management should be comprised and accounted for. On the one hand, the animal health must be preserved and an efficient diagnostic approach is needed to reach a reliable diagnosis on farm and individual level, and on the other hand, the continuous monitoring of newly emerging virus strains in livestock is crucial to update vaccine design and for public health concerns. In the present cross-sectional study, we included individual (nasal swabs, tracheobronchial swabs) and group samples (environmental samples, oral fluids, udder skin wipes) from different stages of production including sows (breeding, gestation, farrowing unit) suckling piglets and nursery pigs (beginning, mid, end of nursery) on 25 sow farms in Germany with attached nursery considered as enzootically infected with swIAV. Our aim was to evaluate the feasibility of different specimens within different stages of pig production in order to establish optimized sampling strategies for monitoring the population of a holding, for achieving individual diagnosis of live animals, and for virus characterization of swIAV for all age groups available on farrow to feeder (30 kg) farms.

## Methods

### Farm selection and study design

For comparison of sampling materials in different age groups a cross-sectional study was performed. Twenty-five farms suspicious for being enzootically infected with swIAV were included. In detail, those farms had to have recurrent respiratory problems and prior detection of swIAV by RT-qPCR and/or hemagglutination inhibition test, which was confirmed by the herd attending vet. Further inclusion criteria were (i) housing of nursery pigs in on-site nursery units and (ii) housing a minimum of ten sows in each production unit (farrowing, breeding, gestation) and (iii) housing a minimum of three different age groups in the nursery.

The sampling protocol and procedures were approved by the Ethic commission of the LMU Munich, accession number 254-10-02-2021.

The farms were enrolled in the study between March 2021 and February 2022. In total, 16 farrow-to-feeder up to 30 kg, eight farrow-to-finish and one breeding farm were involved in the study. The herd size of the farms ranged from 100 to 7000 sows with a median herd size of 475 sows. The farms were spread across different federal states of Germany.

The cross-sectional design included sampling of sows in different production stages (gestation, breeding and farrowing) and parities (gilts, 2nd -4th parity, sows > 4th parity), suckling piglets and three different age groups in the nursery (beginning, mid and end of nursery period).

For individual sampling nasal swabs (NS) in all age groups and tracheobronchial swabs (TBS) in the nursery were selected. Group sampling materials incorporated surface samples, udder skin wipes (USW) and oral fluids (OF). The surface samples consisted of one sample with direct contact to mouth and nose of the pigs (thereafter designated as contact wipe [CW]) and one dust sample (DW). The calculated sample size for individual samples allows the detection of at least one positive sample assuming a within farm prevalence of 15% with a 95% level of confidence, and assuming a 90% sensitivity, 100% specificity of the RT-qPCR. The sample size of group samples was calculated to detect a within-farm swIAV prevalence of 30% assuming the aforementioned levels of sensitivity, specificity and confidence. All sample size calculations were carried out using Epitools epidemiological calculator (Sergeant ESG. Epitools epidemiological calculators: Ausvet Pty Ltd.;2017. Available at: http://epitools.ausvet.com). Sampling of sows in the different production units was stratified by parity of the sows. In the farrowing unit suckling piglets of selected sows were randomly sampled. In the nursery animals with respiratory signs were preferably included in the sampling procedure.

For individually collected nasal swabs a pool size of 3 to 5 individual samples was chosen. Thusly, in order to assure comparability within age groups we decided to collect 10 nasal swab samples from each age group specific category. For individually collected tracheobronchial swabs from nursery piglets, the desired corresponding sample size comprised 15 sampling units, which were evenly allocated to the respective within nursery age group categories. Tracheobronchial swabs were also investigated as pools consisting of 5 individual samples. For environmental and groups samples, the procedure aimed to detect an assumed prevalence of 30% [25], thusly requiring a minimum sample size of 7, further increased to adjust for a homogeneous representation of different production stages and age groups. The detailed sampling protocol and required sample size according to sampling material and age group is presented in Table 1. On each farm 70 nasal swabs, 10 udder skin wipes, 12 surface samples (6 contact wipes + 6 dust wipes), 12 oral fluids, and 15 tracheobronchial swabs were collected accounting for a total of 109 samples per farm.

**Table 1:** Sample size and specimen collected in the different age groups (* in the farrowing unit contact and dust wipes originated from both sows and suckling piglets) on each farm. In sows in each production group 4 gilts, 3 sows of parity 2nd and 3 sows > 4th parity were selected. In suckling piglets one piglet per litter from each sow in the farrowing unit was chosen

Individual samples					Group samples				
Specimen	**NS**	**TBS**			**CW**	**DW**	**USW**	**OF**	
Sows									
Farrowing	10	-			1*	1*	-	-	
Breeding	10	-			1	1	-	-	
Gestation Total: sows	10 30	-			1 3	1 3	- -	- -	
Suckling piglets (2.-3. woa) Total: suckling piglets	10 10	- -			1* 1*	1* 1*	10 10	- -	
Nursery									
Beginning (4.-6.woa)	10	5			1	1	-	4	
Mid (7.-8.woa)	10	5			1	1	-	4	
End (9.-10.woa) Total: nursery	10 30	5 15			1 3	1 3	- -	4 12	
Total: farm	70	15			6	6		12	

### Sample collection

**Fig. 1:**
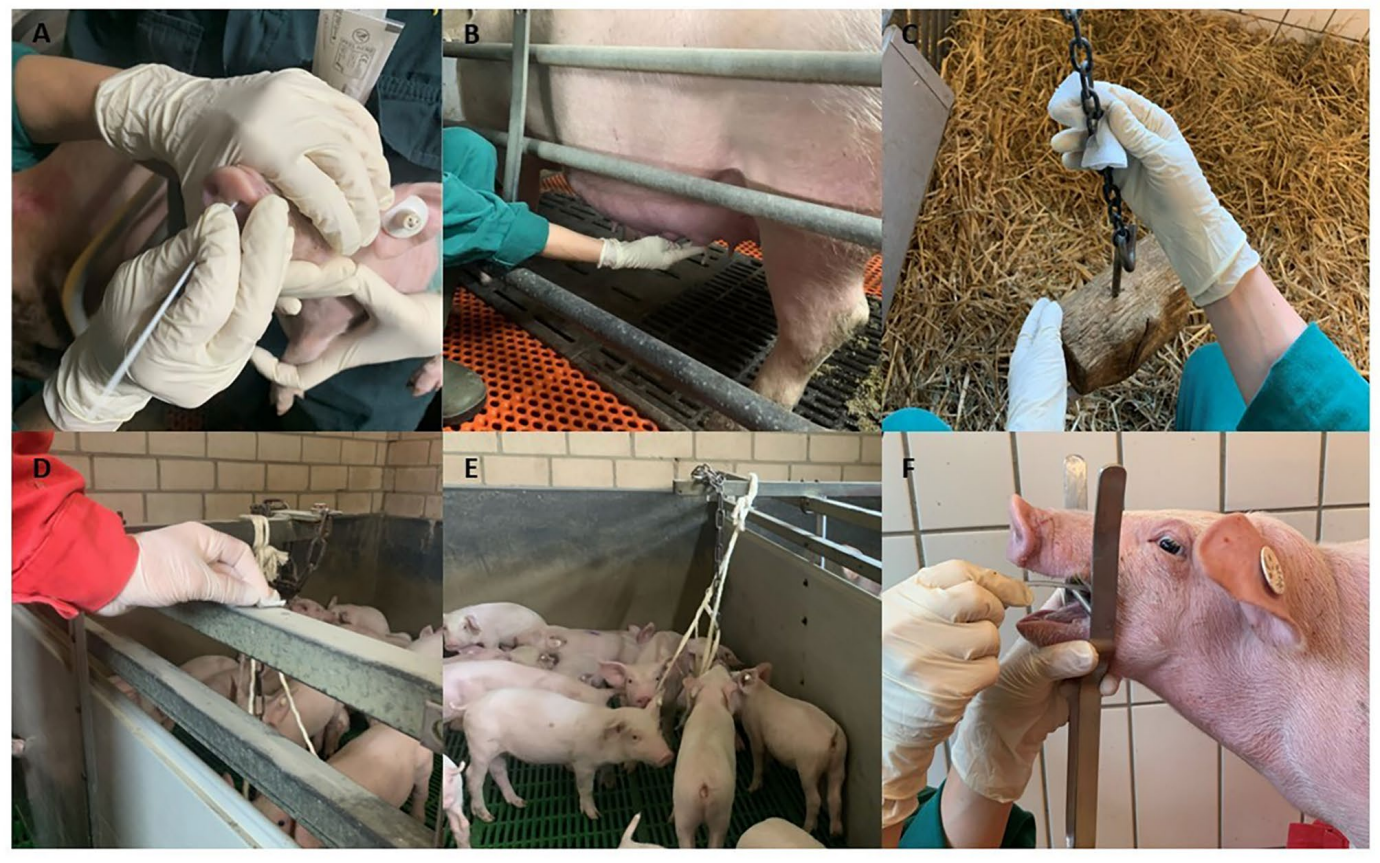
Overview of the different sampling techniques. Collection of **A**: nasal swabs, **B**: sow udder skin wipes, **C**: contact wipes, **D**: dust wipes, **E**: oral fluids, **F**: tracheobronchial swabs

Nasal swabs were collected from individual pigs (Dryswab™ MW113, Check Diagnostics GmbH, Westerau, Germany, for sows and mid/end nursery, Dryswab™ MW112, Check Diagnostics GmbH, Westerau, Germany, for suckling piglets and beginning nursery). Sows were restrained by a snare, whereas suckling piglets and nursery pigs were manually handled and samples were collected by inserting the swab 2–4 cm in both nostrils of each piglet and turning it 360 degrees (Fig. 1A). Nasal swabs of sows and suckling piglets were pooled according to the parity of the sows in pools of 4 or 3 samples, respectively. Nasal swabs of nursery pigs were pooled according to the age group (beginning, mid, end of nursery) in pools of five samples. Nasal swabs were placed into a plastic tube with 2 ml of viral media (Virocult®, Check Diagnostics GmbH, Westerau, Germany).

Collection of udder skin wipes was performed as previously described by Garrido-Mantilla et al. [26]. In brief a 5 × 5 cm sterile gauze pad suspended with NaCl was used for collection of suckling piglets’ secretion at the udder skin (Fig. 1B). After collection samples were placed in plastic tubes with 2 ml of viral media (Virocult®, Check Diagnostics GmbH, Westerau, Germany). At the laboratory udder skin wipes were investigated in pools of 4 or 3 samples according to the parity of the sows.

In each of the six compartments (breeding, gestation, farrowing unit, beginning, mid and end of nursery) two surface samples were collected. Briefly, a 5 × 5 cm sterile gauze pad impregnated with NaCl was used for wiping of surfaces that had direct contact with the mouth and noses of the pigs (e.g. feeders, drinkers, toys, Fig. 1C). Additionally, areas out of the pigs direct range e.g. top of pen separations, feed pipelines, water pipelines were wiped with a 5 × 5 cm sterile gauze pad impregnated with NaCl for collection of dust (Fig. 1D). After collection all surface samples were preserved in 2 ml of viral media (Virocult®, Check Diagnostics GmbH, Westerau, Germany) and examined individually.

Oral fluid collection was performed pen-wise by using the IDEXX Oral Fluid Collection Kit (IDEXX Westbrook, USA). Briefly, an undyed-cotton 3-strand twisted rope was placed into the pen at the height of the pig`s shoulder for 25–30 min, to allow the pig to chew on the rope (Fig. 1E). One rope was used for a maximum of 25 pigs. For extraction of the sample from the rope the wet end was inserted in the supplied plastic bag with the attached tube and manually squeezed. The harvested fluid was suspended in Virocult® media for 1:1 ratio. Each oral fluid sample was investigated individually.

Tracheobronchial swabs were obtained as described previously [27]. Briefly, the pigs in the nursery up to 20 kg were fixed manually by a second person, whereas pigs > 20 kg were restrained using a nose snare. Subsequently a mouth gag was placed between the upper and lower jaw. For TBS collection a sterile catheter (DCT-Nelaton Katheter 40 cm; servoprax GmbH, Wesel, Germany) was used. During inspiration, the catheter was inserted deeply into the trachea until reaching the tracheobronchial split until coughing was provoked (Fig. 1F). Afterwards, the tip of the swab (4–5 cm) was cut off with scissors and transferred to a sterile sample tube containing 4 ml of PBS (Roti®-Cell PBS, Carl Roth GmbH + Co.KG, Karlsruhe, Germany).

Sample tubes were first sent cooled (ice packs) to a diagnostic lab for RNA extraction and swIAV-testing, and later forwarded to the FAO reference center for animal influenza at the Friedrich-Loeffler-Institute (FLI) for identification of swIAV strains.

### Sample preparation

#### Influenza a RT-qPCR

Nasal swabs, oral fluid samples, udder skin wipes, tracheobronchial swabs and environmental samples were collected in 1.5 ml Safe-Lock Tubes (Cat. 72.706.700, Sarstedt, Nümbrecht, Germany) filled with 1 ml of PBS and vortexed. 100–200 µl of the supernatant was then used for isolation of nucleic acid. RNA extraction was done according to the manufacturer´s instructions by using either the IndiMag Pathogen Kit (Cat. SP947257) from Indical Bioscience (Leipzig, Germany) on an Indimag 48 (Cat. IN943048S) or by using the NucleoMag®VET Kit (Macherey-Nagel GmbH & Co. KG, Dueren, Germany) on a Biosprint 96 System, enabling semi-automated processing.

A modified generic Matrix (M)-gene specific influenza A virus RT-qPCR was performed in both laboratories according to Spackman [28] on a CFX96 Touch Real-Time PCR Detection System (Hercules, California, USA), using either Quanta qScript XLT One-Step RT qPCR ToughMix (Beverly, Massachusetts, USA) or the AgPath-IDTM One-Step RT-qPCR kit (Thermo Fisher Scientific, USA).

#### Subtyping RT-qPCRs

Influenza A virus positive samples with a Ct-values $$ <$$32 were forwarded to subtyping RT-qPCR. Further characterization of influenza A virus strains was done by detecting the hemagglutinin (HA) and neuraminidase (NA) genes by quantitative real time RT-qPCRs, allowing identification of subtypes/lineages H1av (clade 1 C), H1pdm (clade 1 A), H1hu (clade 1B) and H3, N1, N1pdm, and N2. Viral RNA was further investigated by using recently modified subtype- and lineage-specific HA and NA RT-qPCRs, allowing subtype specific detection of one further human seasonal subtype: H3hu (2004/2005-derived). RT-qPCR conditions (also for modified primers and probes) were used according to Henritzi et al. [19] and Graaf-Rau et al. [29].

### Statistical analysis

All statistical analyses were performed using Stata 17.0 (Stata Statistical Software, College Station, TX, USA). The significance level was set at 0.05.

A farm was considered positive if at least one of the investigated samples regardless of the sampling material was tested positive by RT-qPCR.

Cochran Q test was used to evaluate differences in the proportion of positive results between the different sampling materials. The level of agreement was calculated using Cohen’s kappa coefficient (κ) or if more than two groups were compared Fleiss’ Kappa was used. Agreement was considered poor if κ ≤ 0.2, fair if 0.21 ≤ κ ≤ 0.4, moderate if 0.41 ≤ κ ≤ 0.6, substantial if 0.61 ≤ κ ≤ 0.8 and good if κ > 0.8 (Petrie A, Watson P. Statistics for Veterinary and Animal Science. 3rd ed. Oxford, U.K.: Wiley-Blackwell, 2013). Whenever a statistically significant difference was detected (*p* < 0.05) all pairwise comparisons were evaluated following Bonferroni’s correction (p_corrected_=0.05/n), where n represents the number of tests or comparisons performed. The potential association of the occurrence of a positive RT-qPCR result with the age-group category (sows, nursing piglets, nursery) from which the respective sample was obtained, for each of the sample types collected (namely nasal swabs, udder skin wipes, contact wipes, dust wipes, oral fluids and tracheobronchial swabs) was investigated in equally numbered two-level mixed effect logistic regression models. In all models, random effect terms at farm level were incorporated to account for the within farm dependence of observations.

#### Multivariable analysis

The investigation of the association of the different types of sampling material and the odds of a positive result by RT-qPCR and subtyping RT-qPCR, within each age group (namely sows, suckling piglets, nursery) was performed with the use of multivariable analysis.. Specifically, in each age group two-three leveled mixed effect logistic regression models were employed using as explanatory variables the type of sample material and the specific location or intra-age group category origin of the collected sample, while the RT-qPCR result and the subtyping RT-qPCR result were considered as the two respective dependent variables. Random effect terms at farm and sample level were incorporated to account for the within farm and within sample dependence of results of RT-qPCR or subtyping RT-qPCR in observations in the same farm and animal or group or pool from different collected materials, respectively.

## Results

### Detection of swIAV by RT-qPCR in the 25 enrolled farms

Detection of influenza A virus by RT-qPCR in at least one sample was possible in 20 (80%) out of the 25 enrolled farms. In the remaining five farms swIAV could not be detected by RT-qPCR, however, antibodies against swIAV were measurable by hemagglutination inhibition test and ELISA in all five farms (data not shown).

### Detection of swIAV in different specimen in the 20 RT-qPCR positive farms

**Fig. 2:**
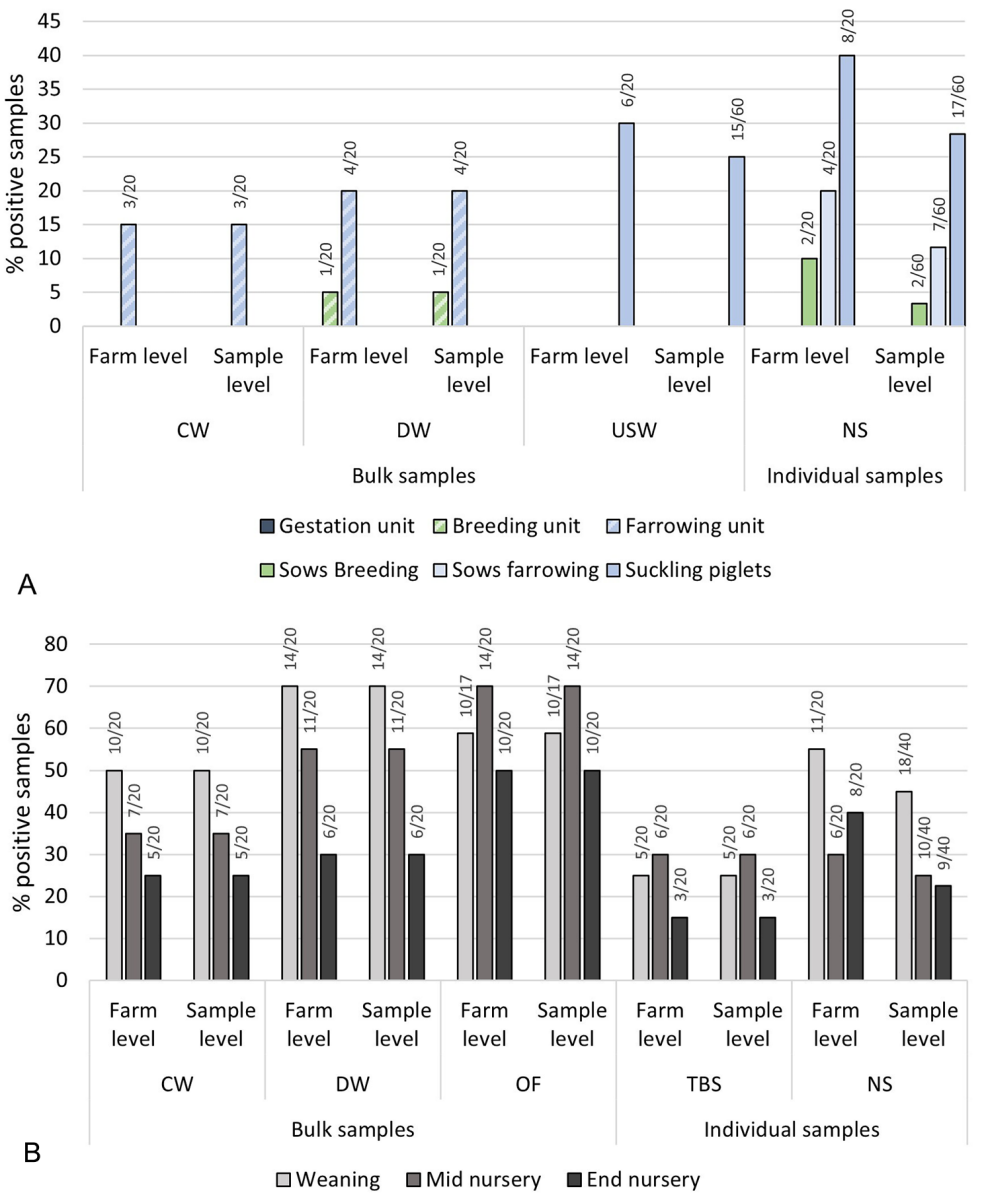
Detection of swIAV by IAV-generic RT-qPCR in different sampling materials in sows and suckling piglets **(A)** and nursery pigs **(B)** on farm level and sample level. A: Percentage of swIAV-RNA positive samples under respect of the used sample material and sampling location. Units apply to environmental samples (contact wipe (CW) + dust wipe (DW)). Sows and suckling piglets apply to animal-based samples (udder skin wipe (USW) + nasal swabs (NS)). In brackets the positive samples out of the total number of investigated samples are presented. B: Percentage of swIAV-RNA positive samples under respect of the used sample material and age group. Next to environmental samples oral fluids (OF) were used as group samples and tracheobronchial swabs (TBS) in addition to NS as individual samples. In brackets the positive samples out of the total number of investigated samples are presented

Subsequently, results are restricted to the 20 RT-qPCR positive farms. A total of 26.1% (218/834) of all available samples, independent of the sampling material, revealed positive RT-qPCR results. Details on positive sampling materials and age groups in each of the 20 RT-qPCR positive farms can be found in supplementary Table 1. The detection rate of swIAV RNA in the different sampling materials is shown in Fig. 2A for sows and suckling piglets and in 2B for nursery pigs. Cochran’s Q test revealed significant differences in the detection rate between the distinct sampling materials in suckling piglets (*p* = 0.034) and in nursery pigs (*p* < 0.001), although the post-hoc test suggested no pair-wise differences between sampling materials (*p* > 0.05) in suckling piglets. In the nursery, pair-wise comparisons have shown significant differences between nasal swabs and dust wipes (*p* = 0.001), nasal swabs and oral fluids (*p* < 0.001), contact wipes and dust wipes (*p* = 0.047), contact wipes and tracheobronchial swabs (*p* = 0.028), contact wipes and oral fluids (*p* < 0.001), dust wipes and tracheobronchial swabs (*p* = 0.001) and tracheobronchial swabs and oral fluids (*p* < 0.001), respectively. No statistically significant differences were observed between nasal swabs and contact wipes (*p* = 0.89), nasal swabs and tracheobronchial swabs (*p* = 1.00) and dust wipes and oral fluids (*p* = 0.23). In sows, no significant differences in the detection rate between the sampling materials could be shown according to Cochran’s Q test, however pairwise comparison revealed significant differences between contact wipes and dust wipes (*p* = 0.043).

### Probability of swIAV detection varies with sample matrix

The probability of sample materials testing positive for swIAV by RT-qPCR was investigated through a multivariable analysis, within each age group, using equally numbered three-level mixed effect logistic regression models. Differences in RT-qPCR detection rate between the different sampling materials are presented in Table 2. In suckling piglets, nasal swabs (*p* = 0.007) and udder skin wipes (*p* = 0.036) showed a significant higher odds of detecting swIAV by RT-qPCR compared to contact wipes, whereas in the nursery the odds to detect swIAV by RT-qPCR were significantly higher in group samples (dust wipes vs. nasal swabs (*p* < 0.001); dust wipes vs. tracheobronchial swabs (*p* < 0.001) contact wipes vs. tracheobronchial swabs (*p* = 0.002); oral fluids vs. nasal swabs (*p* < 0.001) and oral fluids vs. tracheobronchial swabs (*p* < 0.001)) compared to individual samples with the exception of the comparison between contact wipes and nasal swabs (*p* = 0.181).

**Table 2:** Odds ratio (OR) of finding a positive result by sample type using a multivariable analysis, accounting for the within pooled sample and farm-level dependence of observations compared to the respective sampling materials as baseline

Age group	Sample type	Reference	OR with 95%CI	*p*-value
Sows	CW NS	DW	2.76 (0.85; 8.98) 2.76 (0.85; 8.98)	*p* = 0.091 *p* = 0.091
	NS	CW	1.00 (0.29; 3.43)	*p* = 1.000
Suckling piglets	NS	CW	9.44 (1.86; 48)	*p* = 0.007
	USW DW		5.13 (1.12; 23.53) 2.34 (0.52; 10.51)	*p* = 0.036 *p* = 0.266
	DW USW	NS	0.25 (0.05; 1.15) 0.57 (0.13; 2.51)	*p* = 0.075 *p* = 0.461
	DW	USW	0.43 (0.097; 1.92)	*p* = 0.270
Nursery	DW CW OF	NS	5.92 (2.67; 13.09) 1.68 (0.78; 3.59) 13.52 (5.70; 32.09)	*p* < 0.001 *p* = 0.181 *p* < 0.001
	TBS		0.48 (0.21; 1.07)	*p* = 0.073
	CW	TBS	3.50 (1.56; 7.83)	*p* = 0.002
	DW		12.32 (5.25; 28.13)	*p* < 0.001
	OF		28.17 (11.03; 71.92)	*p* < 0.001
	DW	CW	3.52 (1.65; 7.55)	*p* = 0.001
	OF		8.05 (3.54;18.35)	*p* < 0.001
	OF	DW	2.28 (1.06; 4.91)	*p* = 0.034

### Level of agreement between different specimen

The agreement concerning the detection of swIAV RNA between the different sampling materials resulted fair for nursery pigs (Scott/Fleiss’ κ = 0.392; *p* < 0.001) and moderate for suckling piglets (Scott/Fleiss’ κ = 0.588; *p* < 0.001) and sows (Scott/Fleiss’ κ = 0.483; *p* < 0.001). The highest agreement in both sows and suckling piglets was observed between contact wipes and dust wipes, whereas in the nursery nasal swabs and contact wipes showed the highest agreement. The agreement between the different sampling materials within each age group is shown in Table 3.

**Table 3:** Scott/Fleiss’s kappa coefficient (κ) for the agreement of different sampling materials at sample level for the binary variable (positive or negative result of diagnosis of influenza A) within age groups. Whenever a statistically significant association was detected (*p* < 0.05) all pairwise comparisons were evaluated following Bonferroni’s correction (p_corrected_=0.05/n), where n represents the number of tests or comparisons performed

		Sows		Suckling piglets			Nursery			
Material		CW	DW	CW	DW	USW	CW	DW	OF	TBS
NS	kappa (*p*-value)	0.298 (< 0.001)	0.377 (< 0.001)	0.330 (0.003)	0.504 (< 0.001)	0.744 (< 0.001)	0.647 (< 0.001)	0.391 (< 0.001)	0.360 (< 0.001)	0.434 (< 0.001)
CW	kappa (*p*-value)	-	0.733 (< 0.001)	-	0.827 (< 0.001)	0.487 (< 0.001)	-	0.438 (< 0.001)	0.423 (< 0.001)	0.533 (< 0.001)
DW	kappa (*p*-value)	-	-	-	-	0.666 (< 0.001)	-	-	0.457 (< 0.001)	0.181 (0.008)
OF	kappa (*p*-value)	-	-	-	-	-	-	-	-	0.128 (0.023)

### Likelihood of subtyping swIAV strains by multiplex RT-qPCR depends on sample matrix

**Fig. 3:**
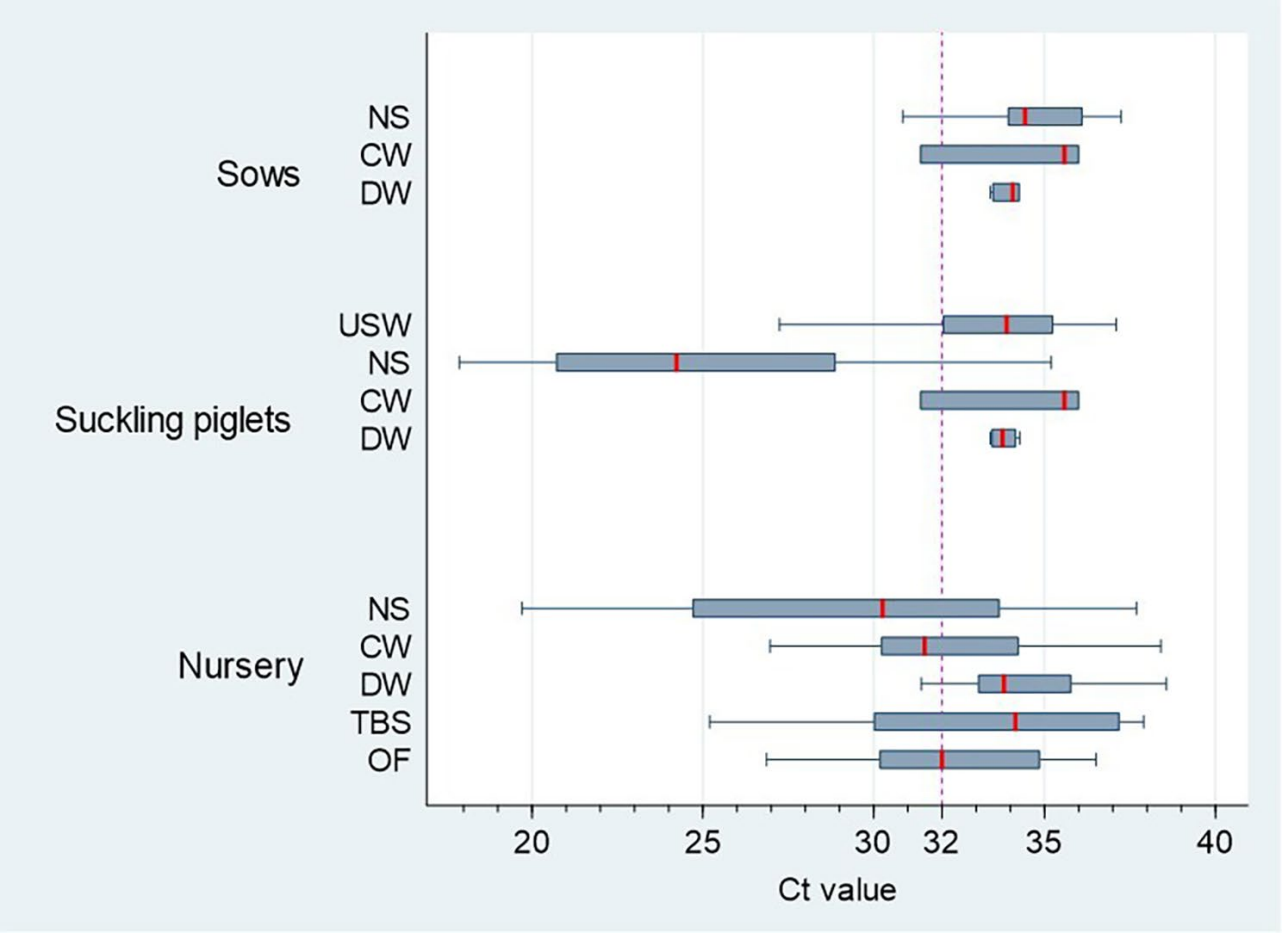
Whisker plots of influenza A virus RT-qPCR cycle threshold (Ct) positive values by age group and sample type

The distribution of RT-qPCR positive Ct-values for each specimen and age group are depicted in Fig. 3.

Based on previous publications and due to lower sensitivity of subtype- and lineage-specific RT-qPCRs compared to the generic IAV RT-qPCR used for initial diagnosis [19, 29], samples with Ct-values of < 32 were selected for subtyping. In total 48.6% (*n* = 106/218) of the positive samples had Ct-values < 32. Of these, subtyping was successful in 97.2% (*n* = 103/106). Referring to the sample type, 66.7% (*n* = 42/63) of the positive nasal swab pools, 52% (*n* = 13/25) of positive contact wipes, 2.8% (*n* = 1/36) of positive dust wipes, 20% (*n* = 3/15) of positive udder skin wipes, 52.9% (*n* = 36/68) of positive oral fluids and 28.6% (*n* = 4/14) of positive tracheobronchial samples had Ct-values$$ <$$32, respectively.

According to multivariable analysis, nasal swabs showed a significantly higher odds of having Ct-values $$ <$$32 compared to dust wipes (*p* < 0.001), contact wipes (*p* = 0.026), udder skin wipes (*p* = 0.001) and tracheobronchial swabs (*p* < 0.001) (Table 4). Interestingly, dust samples showed the lowest probability of detecting samples with Ct-value results $$ <$$32.

**Table 4:** Odds ratios (OR) of finding a sample with Ct-value $$ <$$32 by sample type using a multivariable analysis, accounting for sample type, age group and within farm-level dependence of observations compared to the respective sampling materials as baseline

Sample type	Reference	OR with 95%CI	*p*-value
NS CW	DW	144.03 (26.93; 770.23) 53.77 (10.71; 269.85)	*p* < 0.001 *p* < 0.001
OF		71.41 (13.27; 384.36)	*p* < 0.001
TBS		13.42 (2.38; 75.58)	*p* = 0.003
NS CW	USW	18.73 (3.27; 107.34) 7 (1.22; 40.18)	*p* = 0.001 *p* = 0.029
OF DW TBS		9.28 (1.42; 60.52) 0.13 (0.02 1.09) 1.74 (0.25; 12.34)	*p* = 0.020 *p* = 0.060 *p* = 0.577
NS OF CW	TBS	10.73 (3.05; 37.70) 5.32 (1.57; 17.97) 4 (1.26; 12.73)	*p* < 0.001 *p* = 0.007 *p* = 0.019
CW OF	NS	0.05 (0.01; 0.03) 0.49 (0.21;1.20)	*p* = 0.026 *p* = 0.119
OF	CW	1.33 (0.57;3.10)	*p* = 0.512

In all 20 swIAV positive farms, one or more subtypes could be identified. The subtypes detected in this study were H1avN1, H1avN2, H1pdmN1, H1pdmN2, H1huN1 and H1huN2. The lineage differentiation in the H1 subtype indicates the original sources of the hemagglutinin (HA) as avian (av), human (hu) or human pandemic (pdm). They correspond to the phylogenetically defined clades 1 C (av), 1B (hu) and 1 A (pdm) [30]. A stratified overview of the detection of distinct subtypes in the different specimen is shown in Table 5.

**Table 5:** Number of samples detected for each subtype and specimen type

	H1avN1	H1avN2	H1pdmN1	H1pdmN2	H1huN1	H1huN2	Total
NS	26	7	5	4	4	1	47
CW	10	1	0	0	1	0	12
DW	0	0	0	1	0	0	1
USW	2	0	1	0	0	0	3
OF	26	4	0	6	0	0	36
TBS	4	0	0	0	0	0	4
Total	68	12	6	11	5	1	103

In 70% (*n* = 14/20) of the swIAV RT-qPCR positive farms only a single subtype and in 30% (*n* = 6/20) two or more different subtypes could be detected, respectively. In 4 out of the 6 farms with multiple subtypes, distinct subtypes were found in different age groups. In addition, in 3 out of the 6 farms, different subtypes were detected in different sampling materials (supplementary Table 2).

### Likelihood of detection of swIAV and swIAV subtype characterization varies with age group

Suckling piglets were 7.9 times (95% CI: 3.24; 19.28, *p* < 0.001) more likely to have a positive result in nasal swabs compared to sows. Nasal swabs from nursery pigs were almost 9 times (95% CI: 4.07; 19.77, *p* < 0.001) more likely to have a positive result compared to sows. No difference was observed in the odds of positive results from nasal swabs between suckling and nursery piglets (*p* = 0.724). Accounting for all sampling materials, piglets at the beginning and at the mid of the nursery were more likely to have a positive RT-qPCR result compared to piglets at the end of the nursery, by 4.45 times (95%CI: 1.58; 4.50, *p* < 0.001) and 2.67 times (95% CI: 1.36; 7.32, *p* < 0.001), respectively. Suckling piglets were 152.87 times (95% CI: 6.16; 3792.21, *p* = 0.002) and nursery pigs 19.12 times (95%CI: 1.61; 226.85, *p* = 0.019) more likely to yield nasal swabs with Ct-values < 32 compared to sows. However, no statistically significant difference was observed in the odds of a Ct-value results < 32 between suckling piglets and nursery pigs (95% CI: 0.014; 1.13, *p* = 0.065). In addition, no significant difference in the odds of a subtypeable RT-qPCR result was found between beginning and mid nursery piglets (*p* = 0.287), beginning and end nursery piglets (*p* = 0.897) and between mid and end nursery piglets (*p* = 0.406).

## Discussion

Due to (i) its zoonotic character, (ii) the potential to cause severe respiratory disease in swine and (iii) subsequent economic losses, swIAV represents a pathogen of major importance for public health, animal welfare and for the economy of the swine producing industry [3, 11]. Thus, monitoring and surveillance of swIAV is a major task in controlling herd health in pig production. This requires reliable sampling and test procedures of swIAV diagnostic approaches on herd and individual level. In the past, individual nasal swabs investigated by RT-qPCR have been considered as the gold standard for swIAV detection from live pigs [31, 32]. However, particularly in enzootically infected farms with low prevalence scenarios, a high sample size of nasal swabs is required to reliably detect viral RNA [33]. Thus, more cost-efficient and convenient sampling methods with comparable sensitivity have been sought during the last decade. The group-based approach of oral fluid sampling has become very popular because it is less time-consuming and does not require restriction of individual pigs, hence, it is considered a cost-effective and animal friendly diagnostic tool for swIAV detection [34–39]. However, although the diagnostic sensitivity of oral fluids for qualitative swIAV detection was comparable and the duration of viral RNA shedding was longer in group-based oral fluids compared to individual nasal swabs, the difficulty to isolate swIAV from oral fluids displayed a major drawback for surveillance purposes related to strain characterization [34, 37, 40–42]. Moreover, alternative group sampling techniques such as udder skin wipes offer an additional convenient and cheap sample specimen to monitor swIAV in sow populations with the potential to isolate the virus [26]. Also, surface samples can be regarded as a non-invasive sampling system for swIAV detection by RT-qPCR, but its shortcomings in isolation of swIAV have to be considered [25, 43, 44]. In addition to choosing the most appropriate sample type, the time of sampling within the production process displays a relevant critical point for diagnosis. From an epidemiological point of view, different age groups should be incorporated in the sampling strategy, as the dynamic of infection depends on the susceptibility of the population strata at risk.

Therefore, in the present study, pigs from different age groups of enzootically infected farms in Germany were included to assess which specimen and age group maximizes the likelihood of (i) retrieving swIAV positive samples by RT-qPCR and (ii) enabling subtyping by RT-qPCR. A cross-sectional design was chosen to reflect the diagnostic approach of veterinarians in the field. The five RT-qPCR negative farms, given the selection criteria for the enrolled farms, a history of swIAV infection and the detection of swIAV by RT-qPCR and/or hemagglutination inhibition test prior to the start of the study as well as the results of the hemagglutination inhibition test (data not shown), highlight the challenges of direct virus detection in enzootically infected farms. The dynamic of swIAV infections in swine populations is a subject to variation [45]. Thus, although the farms matched the inclusion criteria prior the start of the study the in-herd prevalence in those farms might have been lower than the 15% underlying our sample size calculation with the consequence of a false negative outcome for the farm`s swIAV status. Therefore, in line with results from Lillie-Jaschniski et al. [46] a high sample size or repeated sampling should be considered in enzootically infected herds in order to increase the probability of swIAV detection on farm level. The examinations of the present study revealed significant variations in the swIAV detection and the subtyping rate between the different specimens. Concerning the farrowing unit, contact wipes had significantly lower odds for swIAV detection compared to pooled nasal swabs of suckling piglets and udder skin wipes of the lactating sows. Based on the assumption that positive udders skin wipes are the result of a contamination from suckling piglets during suckling, a longer and more intense interaction of piglets with the udder compared to the environment and enrichment material might explain these differences. Thus, as udder skin wipes are non-invasive and not burdensome for the suckling piglets, udder skin wipes might represent a suitable sampling material for the detection of swIAV but not for further subtyping purposes in the farrowing unit. Comparable results were also obtained by Garrido-Mantilla et al. [26] within a similar smaller scale study in the USA. However, in our investigations the odds to gain a subtypeable result was significant lower for udder skin wipes (20% typeable RT-qPCR results) compared to nasal swabs (94% typeable RT-qPCR results), thusly nasal swabs represented the most suitable specimen for surveillance of swIAV in the farrowing unit. In contrary, a previous study demonstrated the suitability of udder skin wipes for virus isolation [26]. These differences might be attributed to the divergent subtyping methods, stabilizing media, epidemiological situations on farms or the investigation of udder skin wipes in pools as conducted in our examinations. However according to de Lara et al. [47] pooling of up to 3 udder skin wipes for virus isolation does not result in a decrease of sensitivity. Of note, pooling may yield false reassortment results during further virus isolation and thus it is preferable to use individual samples for isolation purposes and sequencing.

Referring to the nursery, oral fluids were the most appropriate specimen for detection of swIAV RNA followed by dust wipes, contact wipes, nasal swabs and the least suitable specimen, tracheobronchial swabs. The suitability of oral fluids for the monitoring of swIAV, particularly in comparison to nasal swabs, has already been reported elsewhere [34, 48]. However, in line with previous studies the collection of oral fluids at the beginning of the nursery was hampered by the lack of interaction of the weaned piglets with the sampling ropes [26, 40]. This might be overcome by previous training of the pigs or the use of attractants [49–52]. Interestingly, and contrary to earlier reports showing shortcomings of oral fluids in identification of swIAV strains, in our investigations the subtyping rate of oral fluids was comparable to those of nasal swabs [26, 34, 42]. This observation might be explained by the different diagnostic approaches as RT-qPCR analysis directly from clinical specimens was applied in our investigations in contrast to other studies using virus isolation. Moreover, we tried to increase the diagnostic sensitivity by adding Virocult® (Check Diagnostics GmbH, Westerau, Germany) as stabilizing media to the collected oral fluid to preserve the samples during transportation. Interestingly, dust wipes showed a high detection rate, only exceeded by oral fluids in our examinations. However, it should be recognized that the results of oral fluids were more consistent over the different investigated age groups in the nursery compared to dust wipes, which is underlined by the higher odds to detect swIAV RNA in oral fluids (please refer to Fig. 1; Table 2) and an only moderate Cohen´s kappa of oral fluids and dust wipes (Table 3) over the whole nursery period. Furthermore, dust samples demonstrated the lowest subtyping rate among all specimen (2.8%), thus making this kind of specimen unsuitable for surveillance of swIAV under the present conditions. Also, previous studies [25] reported the failure to culture swIAV from surface samples of pen railings and door handles resulting either from non-viable virus or low viral loads. In contrast, the subtyping rate of contact wipes resulting from areas exposed to direct contact of pigs, was significantly higher than those of dust samples and comparable to oral fluids. However, the odds for the initial detection of swIAV was lower in contact wipes compared to dust wipes and oral fluids in the nursery. Referring to tracheobronchial swabs, the burdensome sample procedure for animals and human as well as the low swIAV detection rate [53] disqualifies this type of sample as a tool for the detection of enzootically infected herds. However, tracheobronchial swabs might be suitable for detecting acutely diseased pigs as all tracheobronchial swabs positive samples in the nursery originated from pigs with clinical signs (data presented in [54]). This is also supported by a previous study where tracheobronchial swabs showed the highest correlation in terms of Ct-values with lung samples after a controlled challenge [55].

In line with recent German and European reports H1 clade 1 C was predominantly found, whereas H3N2 could not be detected in any of the samples [10, 29, 56, 57]. Interestingly, in farms with co-circulation of multiple strains, pandemic strains could only be detected in nasal swabs. However due to the small number of observations this has to be elucidated in further studies.

In accordance with previous studies sows were the least suitable population for direct detection of swIAV RNA [33, 58]. In our study, no significant difference in swIAV detection and subtyping rate was observed between suckling piglets and nursery pigs but within the nursery, the chance to detect swIAV RNA in the sample materials was higher at the beginning and the mid of the nursery compared to the end of the nursery. Also previous investigations have shown that sampling of weaners increases the likelihood to identify positive animals compared to suckling piglets and older growing pigs (7–9 weeks of age) [46]. Interestingly, the highest percentage of subtypeable samples was found in suckling piglets. However, the detection of dissimilar subtypes in different age groups which is in line with Lillie-Jaschniski et al. [46] emphasizes the need for incorporating different age groups in the sampling strategy.

## Conclusion

The results of our study highlight that veterinarians should focus on nurserypigs and suckling piglets to detect swIAV RNA and characterize swIAV subtypes in herds with an assumed enzootic infection. Group samples, in particular dust samples and oral fluids have proven to be highly effective for an initial screening of swine herds for swIAV. However, dust samples have major drawbacks in characterization of swIAV subtypes. Interestingly, oral fluids and contact wipes showed high subtyping rates by multiplex RT-qPCR. Nevertheless, nasal swabs are still the most reliable specimen for identification of all on-farm circulating strains. Particularly in farms with recurrent influenza A infections veterinarians should integrate suckling piglets as well as several age groups in the nursery in their sampling approach to gather a detailed overview on the circulating strains.

### Electronic supplementary material

Below is the link to the electronic supplementary material.

Supplementary Material 1

## Data Availability

No datasets were generated or analysed during the current study.

## Abbreviations

Av: avian
Ct: Cycle threshold
CW: contact wipe
DW: dust wipe
ELISA: Enzym linked immunosorbent Assay
FAO: Food and Agriculture Organization
FLI: Friedrich-Loeffler-Institute
HA: hemagglutinin
HI: hemagglutinin inhibition
Hu: human
LMU: Ludwig-Maximilians-Universität
NaCl: sodium chloride
NGS: next generation sequencing
NS: nasal swab
OF: oral fluid(s)
OR: Odds ratio
PBS: Phosphate buffered saline
Pdm: human pandemic
RNA: ribonucleic acid
RT-qPCR: real-time quantitative PCR
swIAV: swine influenza A virus
TBS: tracheobronchial swabs
USW: Udder skin wipes
Woa: weeks of age
