# Supplementary material for: Influenza surveillance in pigs: balancing act between broad diagnostic coverage and specific virus characterization
Source: Porcine Health Manag. 2024 May 19;10:19. doi: 10.1186/s40813-024-00367-9 (PMC11104006; doi:10.1186/s40813-024-00367-9)
Supplement: Supplementary file 1 — Supplementary Material 1 [file 40813_2024_367_MOESM1_ESM.docx]

**Table S1** SwIAV positive age groups and sampling materials on farm level in the 20 RT-qPCR positive farms.

| **Farm ID** | **Farm size (number of sows)** | **Farrowing** | | | | | **Breeding / Gestation** | | | **Nursery** | | | | | |
| --- | --- | --- | --- | --- | --- | --- | --- | --- | --- | --- | --- | --- | --- | --- | --- |
|  |  | **Positivity rate n= x/y (%)** | | | | | **Positivity rate n= x/y (%)** | | | **Positivity rate n= x/y (%)** | | | | | |
|  |  | **Suckling piglets NS (%)** | **CW (%)** | **DW (%)** | **USW (%)** | **Sows NS (%)** | **NS**  **(%)** | **CW (%)** | **DW (%)** | **NS**  **(%)** | **CW**  **(%)** | **DW**  **(%)** | **OF  (%)** | **TBS**  **(%)** | |
| 1 | 7000 | **1** | | | | | 0 | | | 0 | | | | | |
|  |  | 2/3 (66.7) | 1/1 (100) | 1/1 (100) | 2/3 (66.7) | 0/3 (0) | 0/6 (0) | 0/2 (0) | 0/2 (0) | 0/6  (0) | 0/3  (0) | 0/3  (0) | 0/12  (0) | 0/3  (0) | |
| 2 | 170 | **1** | | | | | 0 | | | **1** | | | | | |
|  |  | 3/3 (100) | 0/1 (0) | 0/1 (0) | 3/3 (100) | 0/3  (0) | 0/6  (0) | 0/2 (0) | 0/2 (0) | 2/6 (33.3) | 1/3 (33.3) | 1/3 (33.3) | 3/12 (25) | 0/3  (0) | |
| 3 | 1300 | 0 | | | | | 0 | | | **1** | | | | | |
|  |  | 0/3  (0) | 0/1 (0) | 0/1 (0) | 0/3 (0) | 0/3 (0) | 0/6 (0) | 0/2 (0) | 0/2 (0) | 2/6 (33.3) | 1/3 (33.3) | 1/3 (33.3) | 4/12 (33.3) | 1/3 (33.3) | |
| 4 | 500 | 0 | | | | | 0 | | | **1** | | | | | |
|  |  | 0/3  (0) | 0/1 (0) | 0/1 (0) | 0/3  (0) | 0/3  (0) | 0/6  (0) | 0/2 (0) | 0/2 (0) | 5/6 (83.3) | 2/3 (66.7) | 3/3 (100) | 8/12  (66.7) | 2/3 (66.7) | |
| 5 | 240 | **1** | | | | | 0 | | | 0 | | | | | |
|  |  | 2/3 (66.7) | 0/1 (0) | 0/1 (0) | 0/3  (0) | 1/3 (33.3) | 0/6  (0) | 0/2 (0) | 0/2 (0) | 0/6  (0) | 0/3  (0) | 0/3  (0) | 0/12  (0) | 0/3  (0) | |
| 6 | 330 | 0 | | | | | 0 | | | **1** | | | | | |
|  |  | 0/3  (0) | 0/1 (0) | 0/1 (0) | 0/3  (0) | 0/3  (0) | 0/6 (0) | 0/2 (0) | 0/2 (0) | 2/6 (33.3) | 1/3 (33.3) | 1/3 (33.3) | 5/11 (45.5) | 0/3 (0) | |
| 7 | 5000 | **1** | | | | | **1** | | | **1** | | | | | |
|  |  | 3/3 (100) | 0/1 (0) | 1/1 (100) | 3/3 (100) | 2/3 (66.7) | 1/6 (16.7) | 0/2 (0) | 0/2 (0) | 0/6 (0) | 0/3 (0) | 2/3 (66.7) | 1/11 (9.1) | 0/3 (0) | |
| 8 | 450 | 0 | | | | | 0 | | | **1** | | | | | |
|  |  | 0/3  (0) | 0/1 (0) | 0/1 (0) | 0/3 (0) | 0/3 (0) | 0/6 (0) | 0/2 (0) | 0/2 (0) | 3/6 (50) | 2/3 (66.7) | 3/3 (100) | 10/12 (83.3) | 1/3 (33.3) | |
| 9 | 240 | 0 | | | | | 0 | | | **1** | | | | | |
|  |  | 0/3  (0) | 0/1 (0) | 0/1 (0) | 0/3 (0) | 0/3 (0) | 0/6 (0) | 0/2 (0) | 0/2 (0) | 4/6 (66.7) | 2/3 (66.7) | 2/3 (66.7) | 4/8 (50) | 2/3 (66.7) | |
| 10 | 1000 | 0 | | | | | 0 | | | **1** | | | | | |
|  |  | 0/3  (0) | 0/1 (0) | 0/1 (0) | 0/3 (0) | 0/3 (0) | 0/6 (0) | 0/  (0) | 0/2 (0) | 1/6 (16.7) | 1/3 (33.3) | 1/3 (33.3) | 4/12 (33.3) | 1/3 (33.3) | |
| 11 | 562 | 0 | | | | | 0 | | | **1** | | | | | |
|  |  | 0/3 (0) | 0/1 (0) | 0/1 (0) | 0/3 (0) | 0/3 (0) | 0/6 (0) | 0/2 (0) | 0/2 (0) | 0/6 (0) | 0/3 (0) | 1/3 (33.3) | 0/12 (0) | 0/3 (0) | |
| 12 | 1600 | **1** | | | | | 0 | | | **1** | | | | | |
|  |  | 1/3 (33.3) | 0/1 (0) | 0/1 (0) | 0/3 (0) | 0/3 (0) | 0/6 (0) | 0/2 (0) | 0/2 (0) | 3/6 (50) | 3/3 (100) | 2/3 (66.7) | 5/12 (41.7) | 3/3 (100) | |
| 13 | 2960 | 0 | | | | | 0 | | | **1** | | | | | |
|  |  | 0/3 (0) | 0/1 (0) | 0/1 (0) | 0/3 (0) | 0/3 (0) | 0/6 (0) | 0/2 (0) | 0/2 (0) | 3/6 (50) | 2/3 (66.7) | 1/3 (33.3) | 7/12 (58.3) | 0/3 (0) | |
| 14 | 2700 | 0 | | | | | 0 | | | **1** | | | | | |
|  |  | 0/3 (0) | 0/1 (0) | 0/1 (0) | 0/3 (0) | 0/3 (0) | 0/6 (0) | 0/2 (0) | 0/2 (0) | 2/6 (33.3) | 1/3 (33.3) | 2/3 (66.7) | 2/8 (25) | 0/3 (0) | |
| 15 | 4200 | 0 | | | | | **1** | | | **1** | | | | | |
|  |  | 0/3 (0) | 0/1 (0) | 0/1 (0) | 0/3 (0) | 0/3 (0) | 0/6 (0) | 0/2 (0) | 1/2 (50) | 4/6 (66.7) | 2/3 (66.6) | 3/3 (100) | 7/8 (87.5) | 2/3 (66.7) | |
| 16 | 430 | **1** | | | | | **1** | | | **1** | | | | | |
|  |  | 2/3 (66.7) | 0/1 (0) | 0/1 (0) | 2/3 (66.7) | 1/3 (33.3) | 1/6 (16.7) | 0/2 (0) | 0/2 (0) | 1/6 (16.7) | 0/3 (0) | 1/3 (33.3) | 2/8 (25) | 0/3 (0) | |
| 17 | 230 | 0 | | | | | 0 | | | **1** | | | | | |
|  |  | 0/3 (0) | 0/1 (0) | 0/1 (0) | 0/3 (0) | 0/3 (0) | 0/6 (0) | 0/2 (0) | 0/2 (0) | 0/6 (0) | 0/3 (0) | 2/3 (66.7) | 3/11 (27.3) | 0/3 (0) | |
| 18 | 110 | **1** | | | | | 0 | | | **1** | | | | | |
|  |  | 3/3 (100) | 1/1 (100) | 1/1 (100) | 2/3 (66.7) | 3/3 (100) | 0/6 (0) | 0/2 (0) | 0/2 (0) | 1/6 (16.7) | 0/3 (0) | 2/3 (66.7) | 4/9 (44.4) | 1/3 (33.3) | |
| 19 | 1000 | **1** | | | | | 0 | | | **1** | | | | | |
|  |  | 1/3 (33.3) | 1/1 (100) | 1/1 (100) | 3/3 (100) | 0/3 (0) | 0/6 (0) | 0/2 (0) | 0/2 (0) | 2/6 (33.3) | 1/3 (33.3) | 1/3 (33.3) | 5/11 (45.5) | 0/3 (0) | |
| 20 | 100 | 0 | | | | | 0 | | | **1** | | | | | |
|  |  | 0/3 (0) | 0/1 (0) | 0/1 (0) | 0/3 (0) | 0/3 (0) | 0/6 (0) | 0/2 (0) | 0/2 (0) | 2/6 (33.3) | 3/3 (100) | 2/3 (66.7) | 9/12 (75) | 1/3 (33.3) | |
| Total |  | 17/60 | (3/20) | (4/20) | 15/60 | (7/60) | 2/120 | 0/40 | (1/40) | 37/120 | (22/60) | (31/60) | (83/217) | | (14/60) |

## Table S2 Subtypes found by RT-qPCR in the different sampling materials and age groups.

| **Farm ID** | **Subtype** | **Age group, material** | | | | | | | | | |
| --- | --- | --- | --- | --- | --- | --- | --- | --- | --- | --- | --- |
|  |  | **Sows** | | **Suckling piglets** | | **Beginning Nursery** | | **Mid Nursery** | | **End Nursery** | |
| 1 | H1avN2 | 0 | - | 1 | NS | 0 | - | 0 | - | 0 | - |
| 2 | H1av/H1huN1 | 0 | - | 1 | NS | 1 | NS, CW, OF | 0 | - | 0 | - |
|  | H1av/H1huN2 | 0 | - | 1 | NS | 1 | CW1, OF | 0 | - | 0 | - |
| 3 | H1pdmN2 | 0 | - | 0 | - | 1 | NS | 0 | - | 0 | - |
|  | H1avNx | 0 | - | 0 | - | 1 | NS | 0 | - | 0 | - |
| 4 | H1avN2 | 0 | - | 0 | - | 1 | NS | 0 | - | 0 | - |
|  | H1pdmN2 | 0 | - | 0 | - | 0 | - | 0 | - | 1 | NS |
|  | H1avN1 | 0 | - | 0 | - | 0 | - | 1 | OF | 0 | - |
| 5 | H1avN2 | 0 | - | 1 | NS | 0 | - | 0 | - | 0 | - |
| 6 | H1pdmN1 | 0 | - | 0 | - | 1 | NS | 0 | - | 0 | - |
|  | H1pdmN2 | 0 | - | 0 | - | 1 | NS | 0 | - | 0 | - |
|  | H1avN2 | 0 | - | 0 | - | 1 | OF | 0 | - | 0 | - |
| 7 | H1pdmN1 | 0 | - | 1 | NS, USW | 0 | - | 0 | - | 0 | - |
| 8 | H1pdmN2 | 0 | - | 0 | - | 1 | NS, OF | 1 | OF, DW | 1 | OF |
| 9 | H1avN1 | 0 | - | 0 | - | 1 | NS, TBS | 1 | NS, CW, OF | 0 | - |
| 10 | H1avN1 | 0 | - | 0 | - | 0 | - | 1 | NS, OF | 0 | - |
| 11 | H1avN1 | 0 | - | 0 | - | 0 | - | 0 | - | 1 | DW |
| 12 | H1avN2 | 0 | - | 1 | NS | 0 | - | 0 | - | 0 | - |
|  | H1avN1 | 0 | - | 0 | - | 1 | SW1 | 1 | NS, TBS, OF | 1 | NS, CW, TBS, OF |
| 13 | H1avN2 | 0 | - | 0 | - | 1 | NS | 0 | - | 0 | - |
|  | H1avN1 | 0 | - | 0 | - | 0 | - | 1 | CW, OF | 1 | NS, OF |
| 14 | H1avN1 | 0 | - | 0 | - | 1 | CW | 0 | - | 1 | NS |
| 15 | H1avN1 | 0 | - | 0 | - | 0 | - | 1 | NS, CW, TBS, OF | 1 | NS, TBS, OF |
| 16 | H1avN1 | 0 | - | 1 | NS | 0 | - | 0 | - | 0 | - |
| 17 | H1avN1 | 0 | - | 0 | - | 0 | - | 1 | OF | 0 | - |
| 18 | H1avN1 | 0 | - | 1 | NS, USW, CW | 1 | NS | 0 | - | 0 | - |
| 19 | H1avN1 | 0 | - | 0 | - | 1 | CW, OF, NS | 1 | OF | 0 | - |
| 20 | H1avN1 | 0 | - | 0 | - | 0 | - | 1 | NS | 1 | CW, TBS, OF |
